# Polygenic Risk of Prediabetes, Undiagnosed Diabetes, and Incident Type 2 Diabetes Stratified by Diabetes Risk Factors

**DOI:** 10.1210/jendso/bvad020

**Published:** 2023-01-30

**Authors:** Xiaonan Liu, Jennifer A Collister, Lei Clifton, David J Hunter, Thomas J Littlejohns

**Affiliations:** Nuffield Department of Population Health, University of Oxford, Oxford, Oxfordshire OX3 7LF, UK; Nuffield Department of Population Health, University of Oxford, Oxford, Oxfordshire OX3 7LF, UK; Nuffield Department of Population Health, University of Oxford, Oxford, Oxfordshire OX3 7LF, UK; Nuffield Department of Population Health, University of Oxford, Oxford, Oxfordshire OX3 7LF, UK; Department of Epidemiology, Harvard T.H. Chan School of Public Health, Boston, MA 02115, USA; Nuffield Department of Population Health, University of Oxford, Oxford, Oxfordshire OX3 7LF, UK

**Keywords:** BMI, family history, polygenic risk and diabetes

## Abstract

**Context:**

Early diagnosis of type 2 diabetes is crucial to reduce severe comorbidities and complications. Current screening recommendations for type 2 diabetes include traditional risk factors, primarily body mass index (BMI) and family history, however genetics also plays a key role in type 2 diabetes risk. It is important to understand whether genetic predisposition to type 2 diabetes modifies the effect of these traditional factors on type 2 diabetes risk.

**Objective:**

This work aimed to investigate whether genetic risk of type 2 diabetes modifies associations between BMI and first-degree family history of diabetes with 1) prevalent prediabetes or undiagnosed diabetes; and 2) incident confirmed type 2 diabetes.

**Methods:**

We included 431 658 individuals aged 40 to 69 years at baseline of multiethnic ancestry from the UK Biobank. We used a multiethnic polygenic risk score for type 2 diabetes (PRS_T2D_) developed by Genomics PLC. Prediabetes or undiagnosed diabetes was defined as baseline glycated hemoglobin greater than or equal to 42 mmol/mol (6.0%), and incident type 2 diabetes was derived from medical records.

**Results:**

At baseline, 43 472 participants had prediabetes or undiagnosed diabetes, and 17 259 developed type 2 diabetes over 15 years follow-up. Dose-response associations were observed for PRS_T2D_ with each outcome in each category of BMI or first-degree family history of diabetes. Those in the highest quintile of PRS_T2D_ with a normal BMI were at a similar risk as those in the middle quintile who were overweight. Participants who were in the highest quintile of PRS_T2D_ and did not have a first-degree family history of diabetes were at a similar risk as those with a family history who were in the middle category of PRS_T2D_.

**Conclusion:**

Genetic risk of type 2 diabetes remains strongly associated with risk of prediabetes, undiagnosed diabetes, and future type 2 diabetes within categories of nongenetic risk factors. This could have important implications for identifying individuals at risk of type 2 diabetes for prevention and early diagnosis programs.

Diabetes prevalence has grown substantially in recent decades [1, 2]. In 2021, an estimated 10.5% of the global population was living with diabetes, 90% of whom had type 2 diabetes [1, 2]. This trend is set to continue and is partially attributable to an increase in the prevalence of overweight and obesity, with a high body mass index (BMI) being the strongest risk factor for type 2 diabetes [1–4].

Genetic variation also plays a key role, with type 2 diabetes estimated to be between 30% and 70% heritable [5]. Recent genome-wide association studies have identified hundreds of genetic variants implicated in type 2 diabetes risk [6, 7]. These variants can be summarized in a polygenic risk score (PRS) that provides an overall measure of an individual's inherited predisposition to type 2 diabetes [8]. Consequently, there is growing interest in incorporating genetics into prediction tools to identify high-risk individuals for type 2 diabetes to target for preventive care [9]. Early diagnosis is particularly important, as individuals with undiagnosed type 2 diabetes or prediabetes are at greater risk of developing severe complications and comorbidities [2, 10, 11]. However, PRSs are a relatively recent development and are not currently used in clinical settings or included in screening recommendations [12]. In 2021, the US Preventive Services Task Force (USPSTF) recommended that overweight or obese adults aged 35 to 70 years be screened for prediabetes or type 2 diabetes by clinicians [12]. The USPSTF also recommended that those with a family history of diabetes be considered for screening at younger ages [12]. Given these recommendations and the increasing interest in using PRSs in clinical settings, it is important to understand whether type 2 diabetes PRSs modify the association between BMI or first-degree family history of diabetes and 1) prediabetes or undiagnosed diabetes and 2) future risk of type 2 diabetes. We investigated this in a population-based cohort of approximately 431 000 initially middle-aged participants of multiethnic ancestry from the UK Biobank (UKB) with genotyping data, glycated hemoglobin (HbA_1c_) to determine prediabetes and undiagnosed diabetes at baseline, and follow-up over 15 years to capture incident type 2 diabetes diagnoses.

## Materials and Methods

### Population

The UKB is a population-based study of approximately half a million middle-older aged women and men recruited between 2006 and 2010 across 22 assessment centers in England, Scotland, and Wales [13]. At baseline assessment, data were collected in person via touchscreen questionnaires, a nurse-led verbal interview, a range of physical examinations, and biological samples. Participants consented for UKB to perform ongoing linkage to electronic medical records to collect longitudinal data on incident diseases and death. The UKB received ethical approval from the National Health Service North West Centre for Research Ethics Committee (reference No. 11/NW/0382).

We restricted the study population to participants who were aged 40 to 69 years and had nonmissing core variables (including PRS, HbA_1c_, and BMI at baseline). We excluded participants with prevalent type 1 or 2 diabetes based on 2 sources of data: (1) self-report using a previously published algorithm and (2) hospital inpatient records using International Classification of Diseases codes (ICD-9: 250* and ICD-10: E10*-E14*), with the date of diagnosis preceding or on the date of baseline assessment [14]. Those with implausible HbA_1c_ values, defined as less than 15 mmol/mol (3.5%) or greater than 184 mmol/mol (19.0%), were further excluded. We also excluded participants who were underweight at baseline (BMI < 18.5 kg/m^2^) as lower than recommended weight could reflect underlying health issues.

### Diabetes Polygenic Risk Score, Body Mass Index, and First-degree Family History of Diabetes

We used the standard PRS for type 2 diabetes developed on external multiethnic genome-wide association studies data by Genomics PLC. The PRS was calculated as the sum of the per-variant effect size multiplied by allele dosage, followed by centering and variance-standardization by ancestry. Participants who were sex discordant, or outliers for genotype missingness or heterozygosity were excluded. For the analyses, the PRS was split into quintiles, with a higher quintile indicating a greater risk of developing type 2 diabetes. Hereafter, the type 2 diabetes PRS will be referred to as PRS_T2D_.

BMI (kg/m^2^) was derived from weight (in kilograms) using scales and standing height (in meters) measured during the physical examination with participants categorized as normal (≥ 18.5-< 25), overweight (≥ 25-< 30), and obese (≥ 30) BMI as per World Health Organization (WHO) guidelines. Participants self-reported during the touchscreen questionnaire whether their mother, father, or siblings lived with diabetes and were classified as either having or not having a first-degree family history of diabetes.

### Prediabetes, Undiagnosed Diabetes, and Type 2 Diabetes Outcomes

Prevalent prediabetes or undiagnosed diabetes and incident type 2 diabetes were the primary outcomes. Our composite outcome of prediabetes or undiagnosed diabetes was defined as HbA_1c_ greater than or equal to 42 mmol/mol (6.0%) at baseline [15], in line with accepted thresholds of 42 to 47 mmol/mol (6.0-6.4%) for prediabetes and greater than or equal to 48 mmol/mol (6.5%) for undiagnosed diabetes. HbA_1c_ was measured using nonfasting blood samples collected at baseline assessment using a high-performance liquid chromatography method with Bio-Rad VARIANT II Turbo analyzers [16]. The manufacturer's analytical range was 15 to 184 mmol/mol (3.5%-19.0%). A recent validation study found that HbA_1c_ measured by the UKB were on average lower than HbA_1c_ obtained from primary care records by 2 mmol/mol [17]. Therefore, we calibrated the HbA_1c_ measurements by 0.9696×UKB HbA_1c_ + 3.3595, for the analyses (Supplementary Table S1) [17, 18]. Incident type 2 diabetes (ICD-10: E11*) was derived from hospital inpatient and death registry records.

### Statistical Analysis

In cross-sectional analyses, we used multivariable logistic regression models adjusted for age, sex, BMI, first-degree family history of diabetes, genetic array, and the first 4 principal components of genetic ancestry (provided to the UKB by Genomics PLC) to assess the association between PRS_T2D_ with prediabetes or undiagnosed diabetes. The interaction between PRS_T2D_ and nongenetic risk factors for diabetes was investigated by constructing 2 separate logistic regression models with an added interaction term: PRS_T2D_ × BMI and PRS_T2D_ × first-degree family history of diabetes. From the 2 interaction models, effect estimates of PRS_T2D_ for each category of BMI and first-degree family history of diabetes were obtained. In secondary analyses, we assessed the associations using 2 separate outcomes: prediabetes only (with participants with undiagnosed diabetes excluded) and undiagnosed diabetes only (with participants with prediabetes excluded).

For prospective analyses with incident type 2 diabetes, follow-up time was calculated as the number of years from baseline assessment until date of incident type 2 diabetes, date of death, date of loss to follow-up, or last date of medical record availability in the UKB: September 30, 2021 in England; July 31, 2021 in Scotland; and February 28, 2018 in Wales, whichever came first. We produced 2 age-specific cumulative incidence plots stratified by (1) PRS_T2D_ quintiles and BMI categories; and (2) PRS_T2D_ quintiles and first-degree family history of diabetes. The cumulative incidences were calculated taking into account the competing risk of dying from causes other than type 2 diabetes. For the primary prospective analyses, Cox proportional-hazards models were used to assess the association between PRS_T2D_ and incident type 2 diabetes adjusting for the same covariates included in the cross-sectional analyses. The proportional hazards assumption was visually assessed using scaled Schoenfeld residuals. Interaction terms for the PRS_T2D_ with BMI and first-degree family history of diabetes were entered into separate models, and the effect estimates of PRS_T2D_ for each category were obtained. We conducted further sensitivity analyses for both the cross-sectional and prospective analyses. To check the robustness of results, we additionally adjusted for waist circumference in centimeters (low [≤ 80 for female or ≤ 94 for male]/high [> 80 for female or > 94 for male] [19]), hypertension (yes/no), smoking status (never, previous, current), alcohol units per week (none reported, < 5, 5-9, 10-19, 20-29, ≥ 30), weekly physical activity in metabolic equivalent task minutes (≤ 1200, > 1200), Townsend deprivation index (quintiles) (an indicator of socioeconomic status), household income in British pounds sterling (< 18 000, 18 000-30 999, 31 000-51 999, 52 000-100 000, > 100 000), occupation (professional and administrative, skilled trades, services, manual and industrial, other employment, retired, unable to work because of sickness or disability, unemployed/unanswered), education (5: tertiary, 4: postsecondary nontertiary, 2-3: secondary, 1: primary), and UK country of residence (England, Scotland, Wales). Participants with missing data for these covariates were excluded from the sensitivity analyses. To evaluate whether the results differ by ethnicity, we repeated the main analyses in 2 subgroups, restricting to (1) genetically White and (2) non-White ethnic group. The genetically White group includes individuals who self-report as White British and who have very similar ancestral backgrounds according to the population structure [20]. The non-White ethnic group includes individuals from African, Asian, mixed, and other ethnicity.

Finally, we explored the role of central obesity, by investigating the interaction between PRS_T2D_ and waist circumference with prediabetes, undiagnosed diabetes, and incident type 2 diabetes.

All statistical tests were two-tailed, at a 5% statistical significance level. All analyses were performed using R version 4.0.2.

## Results

Of 502 413 participants, a total of 431 658 participants remained after applying the exclusion criteria (see Supplementary Fig. S1 for flowchart) [18]. Of these, 43 472 (10.1%) had prediabetes or undiagnosed diabetes at baseline, and 17 259 (4.0%) developed type 2 diabetes over a median of 12.5 (interquartile range = 11.6-13.2) years of follow-up. Among the 43 472 participants who had prediabetes (n = 38 319) or undiagnosed (n = 5153) diabetes at baseline, 9827 (22.6%) were diagnosed with type 2 diabetes during follow-up. Participants in the highest PRS_T2D_ quintile were more likely to be younger, from non-White ethnic groups, obese, have a first-degree family history of diabetes, and have higher HbA_1c_ at baseline (Table 1).

**Table 1.** Baseline characteristics of 431 658 participants by quintiles of type 2 diabetes polygenic risk score

	Type 2 diabetes PRS					
Characteristic, N (%)	Q1 (N = 86 332)	Q2 (N = 86 331)	Q3 (N = 86 332)	Q4 (N = 86 331)	Q5 (N = 86 332)	Total (N = 431 658)
Age, y						
40-49	20 079 (23.3)	20 297 (23.5)	20 572 (23.8)	20 967 (24.3)	22 343 (25.9)	104 258 (24.2)
50-59	28 763 (33.3)	28 863 (33.4)	28 983 (33.6)	29 042 (33.6)	29 424 (34.1)	145 075 (33.6)
60-69	37 490 (43.4)	37 171 (43.1)	36 777 (42.6)	36 322 (42.1)	34 565 (40.0)	182 325 (42.2)
Women	47 440 (55.0)	47 483 (55.0)	47 197 (54.7)	47 451 (55.0)	48 041 (55.6)	237 612 (55.0)
White British	72 959 (84.5)	73 404 (85.0)	73 249 (84.8)	73 073 (84.6)	72 414 (83.9)	365 099 (84.6)
BMI						
Normal	35 071 (40.6)	30 833 (35.7)	28 861 (33.4)	27 131 (31.4)	24 628 (28.5)	146 524 (33.9)
Overweight	36 002 (41.7)	37 176 (43.1)	37 547 (43.5)	37 627 (43.6)	38 220 (44.3)	186 572 (43.2)
Obese	15 259 (17.7)	18 322 (21.2)	19 924 (23.1)	21 573 (25.0)	23 484 (27.2)	98 562 (22.8)
First-degree family history of diabetes	11 609 (13.4)	14 640 (17.0)	17 177 (19.9)	19 546 (22.6)	24 615 (28.5)	87 587 (20.3)
HbA_1c_ ≥ 42 mmol/mol (6.0%)	4619 (5.4)	6496 (7.5)	8171 (9.5)	10 158 (11.8)	14 028 (16.2)	43 472 (10.1)

Abbreviations: BMI, body mass index; HbA_1c_, glycated hemoglobin; PRS, polygenic risk score; Q, quintile.

In multivariable logistic regression models, higher PRS_T2D_ quintiles were associated with an increased risk of prediabetes or undiagnosed diabetes. The odds ratios (ORs) were 0.58 (95% Confidence Interval [CI], 0.56-0.60), 0.79 (95% CI, 0.77-0.82), 1.26 (95% CI, 1.22-1.30), and 1.81 (95% CI, 1.76-1.87) for quintiles 1, 2, 4, and 5, respectively, compared to quintile 3. A first-degree family history of diabetes and higher BMI were also associated with prediabetes or undiagnosed diabetes (Supplementary Table S2) [18]. There was a statistically significant interaction between PRS_T2D_ and BMI (*P* < .001) but not first-degree family history (*P* = .63) for prediabetes or undiagnosed diabetes (Table 2, and Supplementary Fig. S2) [18]. The odds of having prediabetes or undiagnosed diabetes increased with PRS_T2D_ for all BMI and first-degree family history of diabetes categories. However, when comparing PRS_T2D_ quintile 5 with quintile 3, the strength of association was slightly greater in those overweight (OR = 1.93; 95% CI, 1.85-2.03) or obese (OR = 1.78; 95% CI, 1.69-1.87) than those with normal BMI (OR = 1.64; 95% CI, 1.53-1.75). In secondary analyses using prediabetes (Supplementary Table S3) [18] and undiagnosed diabetes (Supplementary Table S4) [18] as separate outcomes, the direction of the associations remained similar, but compared to the main analyses (which combined them) the effect estimates were generally weaker with prediabetes and stronger with undiagnosed diabetes.

**Table 2.** Logistic regression models investigating the association between type 2 diabetes polygenic risk score and glycated hemoglobin-defined prediabetes or undiagnosed diabetes by BMI and first-degree family history of diabetes status in 431 658 participants

		Type 2 diabetes PRS OR (95% CI)					*P* for interaction
	Cases/Population	Q1	Q2	Q3	Q4	Q5	
**BMI**							
Normal	8114/146 524	0.57 (0.53-0.62)	0.79 (0.73-0.85)	1 (Reference)	1.25 (1.16-1.34)	1.64 (1.53-1.75)	
Overweight	17 479/186 572	0.58 (0.54-0.61)	0.80 (0.75-0.84)	1 (Reference)	1.26 (1.20-1.33)	1.93 (1.85-2.03)	
Obese	17 879/98 562	0.58 (0.54-0.62)	0.79 (0.74-0.84)	1 (Reference)	1.26 (1.19-1.32)	1.78 (1.69-1.87)	< .001*^a^*
**First-degree family history of diabetes**							
No	31 100/344 071	0.58 (0.56-0.61)	0.79 (0.76-0.83)	1 (Reference)	1.27 (1.23-1.32)	1.83 (1.77-1.90)	
Yes	12 372/87 587	0.56 (0.51-0.61)	0.79 (0.74-0.85)	1 (Reference)	1.22 (1.14-1.29)	1.77 (1.67-1.87)	.63*^b^*

Abbreviations: BMI, body mass index; OR, odds ratio; CI, Confidence Interval; PRS, polygenic risk score; Q, quintile.

a Model: outcome ∼ age + sex + BMI + family history + genetic array + first 4 genetic principal components + PRS × BMI.

b Model: outcome ∼ age + sex + BMI + family history + genetic array + first 4 genetic principal components + PRS × family history.

**Figure 1.**
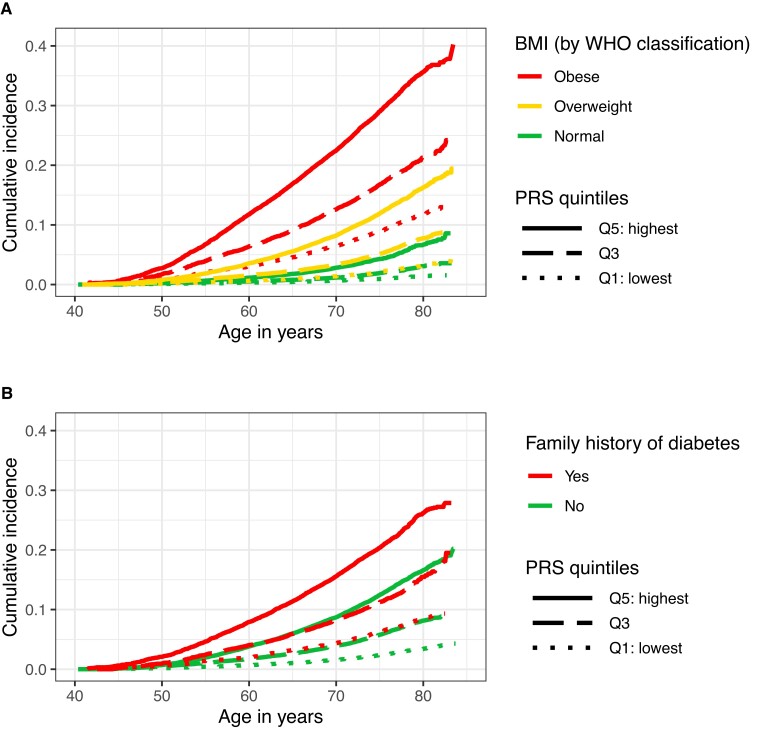
Age-specific cumulative incidence of type 2 diabetes by type 2 diabetes polygenic risk score (PRS) and A, body mass index (BMI) and B, first-degree family history of diabetes in 431 658 participants.

To investigate the potential role of the PRS_T2D_ in earlier-life type 2 diabetes screening, we compared the age-specific cumulative incidence of type 2 diabetes across PRS_T2D_ quintiles and risk factor categories. The age-specific cumulative incidence of type 2 diabetes is increased in each higher PRS_T2D_ quintile within each category of BMI (Fig. 1A) and first-degree family history of diabetes (Fig. 1B). From approximately age 45 years, the absolute difference in cumulative incidence between PRS_T2D_ quintiles is greater in those with a stronger risk of diabetes based on BMI or a first-degree family history of diabetes. Furthermore, those with a normal BMI but in the highest PRS_T2D_ quintile had a similar age-specific incidence of type 2 diabetes to those who were overweight but in the middle PRS_T2D_ quintile. We also observed the age-specific cumulative incidence curves between people with an overweight BMI in the highest PRS_T2D_ quintile were higher than those who were obese in the lowest PRS_T2D_ quintile. Similar findings for first-degree family history of diabetes were observed. For instance, those without a first-degree family history of diabetes in the highest PRS_T2D_ quintile had a similar age-specific incidence of type 2 diabetes compared to those with a first-degree family history of diabetes in the middle PRS_T2D_ quintile.

In the multivariable Cox proportional hazards model, higher PRS_T2D_ quintiles were associated with an increased risk of incident type 2 diabetes. The hazard ratios (HRs) were 0.49 (95% CI, 0.45-0.52), 0.79 (95% CI, 0.75-0.84), 1.31 (95% CI, 1.25-1.37), and 1.97 (95% CI, 1.89-2.06) for quintiles 1, 2, 4, and 5, respectively, compared to quintile 3. A first-degree family history of diabetes and higher BMI were also associated with incident type 2 diabetes (Supplementary Table S2) [18]. Statistically significant interactions were observed between PRS_T2D_ × BMI (*P* < .001) and between PRS_T2D_ × first-degree family history of diabetes (*P* < .001) with risk of incident type 2 diabetes (Table 3, and Supplementary Fig. S3) [18]. A dose-response association between PRS_T2D_ and incident type 2 diabetes was observed within each category of BMI and first-degree family history of diabetes. The strength of the associations were greater in the higher quintiles among participants with a normal or overweight BMI and weaker in obese participants. The effect of PRS_T2D_ was slightly stronger for those without existing first-degree family history of diabetes.

**Table 3.** Cox proportional-hazards models investigating the association between type 2 diabetes polygenic risk score and incident type 2 diabetes by body mass index and first-degree family history of diabetes status in 431 658 participants

		Type 2 diabetes PRS HR (95% CI)					*P* for interaction
	Cases/Population	Q1	Q2	Q3	Q4	Q5	
**BMI**							
Normal	1614/146 524	0.50 (0.41-0.60)	0.80 (0.67-0.95)	1 (Reference)	1.44 (1.24-1.68)	2.21 (1.92-2.56)	
Overweight	6199/186 572	0.42 (0.38-0.47)	0.74 (0.68-0.82)	1 (Reference)	1.36 (1.26-1.47)	2.19 (2.04-2.35)	
Obese	9446/98 562	0.54 (0.49-0.59)	0.82 (0.77-0.89)	1 (Reference)	1.26 (1.18-1.34)	1.80 (1.70-1.91)	< .001*^a^*
**First-degree family history of diabetes**							
No	11 536/344 071	0.46 (0.43-0.50)	0.79 (0.74-0.84)	1 (Reference)	1.34 (1.26-1.41)	2.06 (1.96-2.17)	
Yes	5723/87 587	0.57 (0.51-0.65)	0.80 (0.72-0.88)	1 (Reference)	1.25 (1.15-1.36)	1.81 (1.68-1.95)	< .001*^b^*

Abbreviations: BMI, body mass index; HR, hazard ratio; CI, Confidence Interval; PRS, polygenic risk score; Q, quintile.

a Model: outcome ∼ age + sex + BMI + family history + genetic array + first 4 genetic principal components + PRS × BMI.

b Model: outcome ∼ age + sex + BMI + family history + genetic array + first 4 genetic principal components + PRS × family history.

In a sensitivity analysis including additional adjustments—waist circumference, hypertension, smoking status, alcohol units per week, weekly physical activity, Townsend deprivation index, household income, occupation, education, and UK country of residence—the interaction between PRS_T2D_ and BMI for prediabetes or undiagnosed diabetes was attenuated but the findings for incident type 2 diabetes remained similar to the main findings (Supplementary Tables S5 and S6) [18]. The direction and strength of associations obtained in genetically White (N = 365 099) and non-White populations (N = 66 559) (Supplementary Tables S7-S10) [18] were similar. Similar to the results obtained from BMI, PRS_T2D_ were associated with diabetes outcomes regardless of low or high waist circumference (Supplementary Tables S11 and S12) [18]. Effect modification of waist circumference by PRS_T2D_ was statistically significant for incident type 2 diabetes (*P* = .04) but not for prediabetes or undiagnosed diabetes (*P* = .17).

## Discussion

In this large cohort of approximately 431 000 middle-to-older aged adults, polygenic risk for type 2 diabetes was associated with an increased risk of having prevalent prediabetes or undiagnosed diabetes, and future risk of developing type 2 diabetes over a period of 15 years. The relative associations of having a higher PRS_T2D_ with prediabetes or undiagnosed diabetes were stronger among those with a higher BMI, whereas the relative associations between the PRS_T2D_ and incident type 2 diabetes were stronger among those with a lower BMI. A stronger association was observed with incident type 2 diabetes among those without a first-degree family history of diabetes. Nevertheless, the direction of associations remained consistent within those with a normal, overweight, or obese BMI and those with or without a first-degree family history of diabetes.

BMI is the strongest modifiable risk factor for type 2 diabetes, with a recent meta-analysis of 182 studies finding that each 5-unit increase of BMI was linked with a 72% increased risk of type 2 diabetes [21]. This is reflected in national guidelines for screening and prevention. For example, the US-based USPSTF recommends type 2 diabetes screening among overweight and obese individuals, while the WHO highlights obesity as a key target for type 2 diabetes prevention [2, 12]. First-degree family history of diabetes is also a strong risk factor for type 2 diabetes. Although family history is not modifiable and is a proxy both for genetic and environmental risk factors, it is a valuable marker for identifying individuals at greater risk for type 2 diabetes [2, 22]. In addition to typical sociodemographic characteristics, such as age, sex, and ethnicity, risk scores for type 2 diabetes typically place a strong emphasis both on BMI and first-degree family history of diabetes [23–25].

However, the findings in the present study suggest that genetic risk produces substantial differences both in the relative and absolute risk of type 2 diabetes among individuals classified as “low,” “normal,” or “high” risk based on classic risk factors. In those with a normal BMI, the highest PRS_T2D_ quintile was associated with a more than 60% increase in the risk of having prediabetes or undiagnosed diabetes and more than double the risk in developing incident type 2 diabetes compared to those in the middle PRS_T2D_ quintile. Similarly, in those who were obese, the risk for both outcomes was between 78% and 80% greater in the highest vs middle PRS_T2D_ quintile. The observed overlap between BMI and first-degree family history of diabetes categories in age-specific cumulative incidence curves suggests that PRS_T2D_ would change the absolute risk of individuals across categories of risk factors. For example, the absolute risk of individuals with a normal BMI and PRS_T2D_ in the highest quintile was almost equivalent to those with an overweight BMI and PRS_T2D_ in the middle quintile. We also observed similar findings for waist circumference, a marker of central obesity, and a risk factor for type 2 diabetes independent of overall adiposity [21]. This could have direct implications for decision-making in preventive screening for type 2 diabetes. Individuals who are currently classified as “low” risk for type 2 diabetes based on traditional factors might be suitable for further screening based on their genetic risk. Existing risk prediction tools calculate an individual's risk of type 2 diabetes based on various sociodemographic and lifestyle factors [24, 25]. To understand the potential clinical relevance of our findings, an important next step would be to investigate whether incorporating genetic predisposition to type 2 diabetes into existing tools results in improved risk prediction.

Our findings for PRS_T2D_, BMI, and incident type 2 diabetes were similar to those from the InterAct study of 340 234 participants in the EPIC cohort (n = 12 403 cases), which also observed stronger relative risks of PRS_T2D_ in those with a lower BMI but greater absolute risks in those with a higher BMI [26]. Two separate studies primarily designed to investigate interactions between lifestyle and genetic risk with cardiovascular disease and diabetes found in secondary analyses that BMI and genetic risk for diabetes jointly combined to increase incident diabetes risk [27, 28]. We additionally observed these associations for prediabetes and undiagnosed diabetes as well as investigating the role of first-degree family history for diabetes. The association between PRS_T2D_ and type 2 diabetes was stronger among those without, than with, a first-degree family history of diabetes. One explanation could be that having a family history of diabetes is such a strong type 2 diabetes risk factor that the relative PRS_T2D_-outcome associations are attenuated in this group of individuals. This explanation could also apply to BMI, where weaker relative risks with incident type 2 diabetes were observed in those with a higher BMI.

A major strength of this study is the large sample size in combination with detailed data collection, including genetic, biomarker, and longitudinal linkage to electronic medical records over a long follow-up period. We also calibrated HbA_1c_ as recent findings suggest that the “raw” UKB measurements substantially underestimate the prevalence of prediabetes and undiagnosed diabetes [17]. The main findings of type 2 diabetes remained consistent when including additional adjustment for a range of sociodemographic lifestyle and health-related factors. This study also has several limitations. First, even though our analysis population was multiethnic, the UKB cohort is predominately of White genetic ancestry (83.9%). In the present study, the results were similar both in White and non-White participants; however, replication in larger non-White genetic data sets is still necessary. Second, the ascertainment of incident type 2 diabetes was based on hospital inpatient and death registry records. While these sources demonstrate high accuracy for capturing “true” cases, they underestimate cases captured in other sources, such as primary care records [14]. Therefore, the cumulative incidences of type 2 diabetes presented in the present manuscript are likely to be underestimated. Third, the UKB cohort demonstrates evidence of a healthy volunteer effect, with the BMI of UKB participants on average lower compared to the general population [29]. Fourth, first-degree family history of diabetes was self-reported and did not distinguish between type 1 and 2 diabetes.

We found that increased genetic risk for type 2 diabetes was strongly associated with prediabetes, undiagnosed diabetes, and incident type 2 diabetes regardless of BMI or first-degree family history of diabetes. These findings could have implications for the identification of individuals to target for diabetes-prevention programs and the earlier diagnosis of type 2 diabetes. Investigating whether risk prediction for type 2 diabetes could be enhanced by incorporating genetic risk factors is an important next step.

## Data Availability

Restrictions apply to the availability of some or all data generated or analyzed during this study to preserve patient confidentiality or because they were used under license. The corresponding author will on request detail the restrictions and any conditions under which access to some data may be provided.

## Abbreviations

BMI: body mass index
HbA_1c_: glycated hemoglobin
ICD: International Classification of Diseases
OR: odds ratio
PRS: polygenic risk score
PRS_T2D_: polygenic risk score for type 2 diabetes
UKB: UK Biobank
USPSTF: US Preventive Services Task Force
WHO: World Health Organization
